# *In vitro* Incubation of Platelets with oxLDL Does Not Induce Microvesicle Release When Measured by Sensitive Flow Cytometry

**DOI:** 10.3389/fcvm.2015.00037

**Published:** 2015-11-30

**Authors:** Tine Bo Nielsen, Morten Hjuler Nielsen, Aase Handberg

**Affiliations:** ^1^The Doctoral School in Medicine, Faculty of Medicine, Aalborg University, Aalborg, Denmark; ^2^Department of Clinical Biochemistry, Aalborg University Hospital, Aalborg, Denmark; ^3^Department of Clinical Medicine, Faculty of Medicine, Aalborg University, Aalborg, Denmark

**Keywords:** extracellular vesicles, flow cytometry, CD36, lactadherin, platelets, oxidized low-density lipoprotein

## Abstract

Microvesicles (MVs) are submicron vesicles with sizes of 0.1–1.0 μm in diameter, released from various cell types upon activation or apoptosis. Their involvement in a variety of diseases has been intensively investigated. In blood, platelets are potent MV secretors, and oxidized low-density lipoprotein (oxLDL), a platelet ligand, induces platelet activation and thus potentially MV secretion. This interaction occurs through binding of oxLDL with CD36, located on the platelet membrane. In this study, we investigated the effect of *in vitro* incubation of platelets with oxLDL on MV release. Furthermore, we compared the results obtained when separating MVs larger than 0.5 μm as a measure of results obtained from less sensitive conventional flow cytometers with MVs below the 0.5 μm limit. MV size distribution was analyzed in plasma from 11 healthy volunteers (four females and seven males). MVs were identified as <1 μm and positive for lactadherin binding and cell-specific markers. Platelet-rich plasma (PRP) was incubated without and with oxLDL or LDL (as control) to investigate the impact on platelet activation, evident by release of MVs. Size-calibrated fluorescent beads were used to establish the MV gate, and separate small- and large-size vesicles. CD41^+^ and CD41^+^CD36^+^ MVs increased by six to eightfold in PRP, when left at room temperature, and the presence of cell-specific markers increased. Total MV count was unaffected. Incubations with oxLDL did not increase the MV release or affect the distribution of small- and large-size MVs. We found a large interindividual variation in the fraction of small- and large-size MVs of 73%. In conclusion, we propose that procoagulant activity and activation of platelets induced by interaction of platelet CD36 with oxLDL may not involve release of MVs. Furthermore, our results demonstrate great interindividual variability in size distribution of platelet-derived MVs and thereby stress the importance for generation of standardized protocols for MV quantification by flow cytometry.

## Introduction

Microvesicles (MVs) are submicron vesicles with sizes of 0.1–1.0 μm in diameter. They are released from various cell types as a result of cellular activation or apoptosis (1). In recent years, MVs have received more attention due to their potential involvement in a variety of disease states, such as cancer, inflammatory and autoimmune diseases, cardiovascular disorders, and the metabolic syndrome (2–6). The roles of MVs are diverse. They have been shown to transfer material, in the form of membrane proteins, DNA, RNA, receptors, or cytoplasmic components, from the parent cell to recipient cells in other areas of the body (1).

Platelets are potent MV secretors in blood. Various ligands potentiate platelet activation, which can lead to MV secretion.

Oxidized low-density lipoprotein (oxLDL) known to induce a prothrombotic state activates platelets through binding with CD36 (7, 8), a scavenger receptor abundantly present in the platelet membrane. Studies have shown that when platelet CD36 binds oxLDL, specific intracellular signaling cascades are activated, resulting in platelet activation (9, 10), thus promoting a prothrombotic state and possibly the release of MVs.

Flow cytometry is a widely used method for the detection of MVs originating from different cells or tissues. The method is a powerful tool to detect, quantify, and characterize MVs, but the sensitivity of most instruments is challenged by the small size of the particles and the entailing dim signal. Several studies, performed on conventional flow cytometers, report a detection limit of 0.5 μm (11), which possibly discards a vast amount of the MVs as background noise or debris. The more dedicated flow cytometers allows for detection below 0.5 μm thereby providing access to the small-size MVs (12). The relationship between MVs measured by conventional flow cytometers and the results obtained when measuring within the full MV size range may be important for the interpretation and comparison of new data with older, obtained by less sensitive flow cytometers.

The primary aim of this study was to investigate the impact of *in vitro* incubation of platelets with oxLDL on secretion of platelet-derived MVs (PMVs) and in particular PMVs carrying CD36 measured by a sensitive flow cytometer. The secondary aim was to compare the results obtained when separating MVs larger than 0.5 μm as a measure of results obtained from less sensitive conventional flow cytometers with MVs below the 0.5 μm limit.

## Materials and Methods

### Donors and Blood Collection

Eleven healthy volunteers were included in the study (four females and seven males). According to a medical questionnaire, the participants were free of any overt disease, non-smoking and had not been taking any medication affecting coagulation for the past 10 days. Blood samples were drawn from the antecubital vein with the help of a tourniquet and using a butterfly system with a 21-G needle (SAFETY blood collection set, 21 G, Vacuette, Greiner Bio-one, Kremsmünster, Austria). Blood was collected into 3.2% (0.109M) citrated tubes (Vacuette^®^, 9 mL, Greiner Bio-one, Kremsmünster, Austria), K3E EDTA tubes, and Lithium Heparin (Vacuette^®^, 3 mL, Greiner Bio-one, Kremsmünster, Austria). Biochemical screening was performed on a Cobas^®^ 6000 analyzer (Roche Diagnostics Ltd., Switzerland) and an Advia 2120i Hematology Systems (Siemens, Germany). A Sysmex KX-21N Automated Hematology Analyzer (Sysmex Corporation, Japan) was also used for measuring platelet count in platelet-rich plasma (PRP). The study was conducted in agreement with the Declaration of Helsinki, and the protocol was approved by the local Ethics Committee of North Region Denmark (Reg. Nb. N-20110067). Informed written consent was obtained from all study participants.

### Sample Preparation

**Figure 1:**
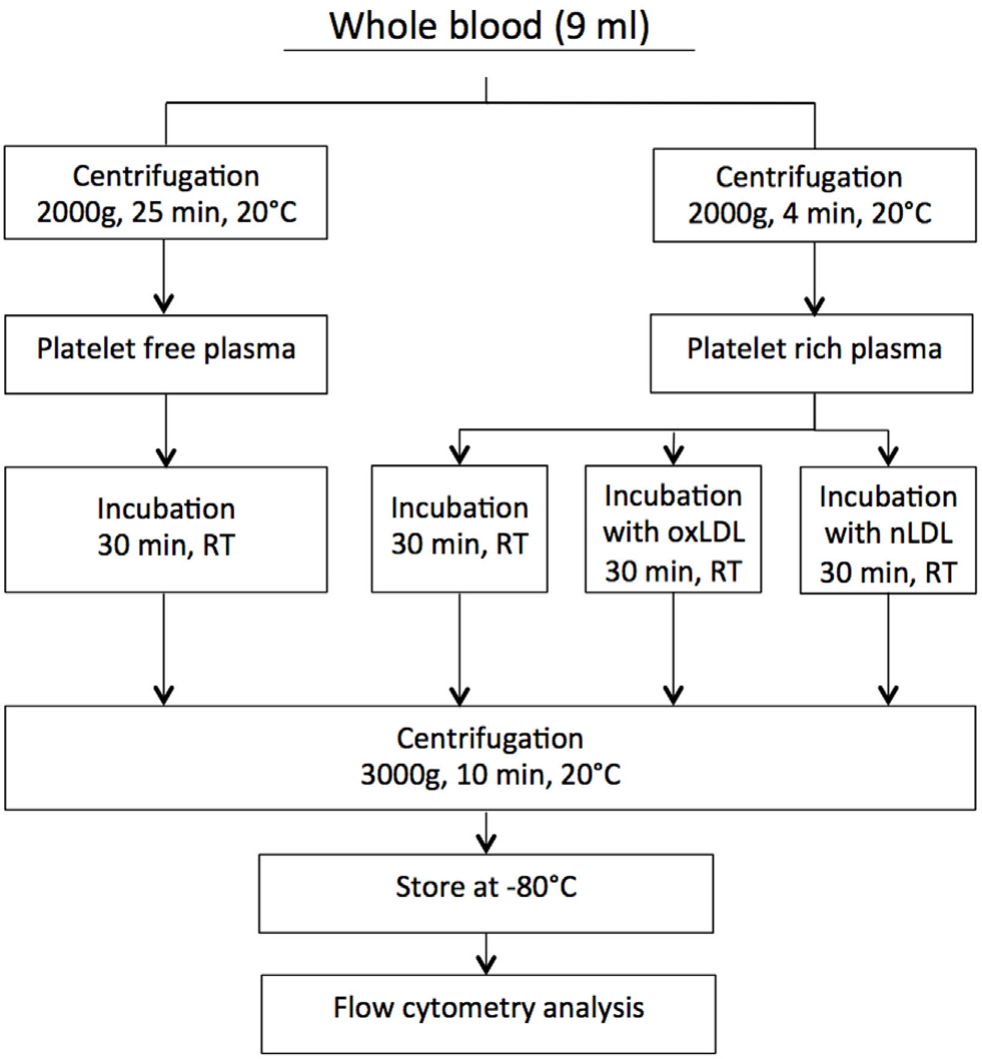
**The centrifugation procedure**.

Citrated blood samples were centrifuged at two different settings to obtain both PRP and platelet-free plasma (PFP). The latter was used as control in the experimental set-up. To obtain PRP, the blood samples were centrifuged at 2000 *g* for 4 min at 20°C, and to obtain PFP, the samples were centrifuged at 2000 *g* for 25 min at 20°C. Autologous PFP was used for adjusting platelet count in PRP to contain 150–200 × 10^−9^/L. Before flow cytometry analyses, all plasma samples were further centrifuged at 3000 *g* for 10 min in order to remove platelets. Since much smaller volumes were centrifuged following incubations. Stoke’s equation was used for calculation of g-force and time to obtain sedimentation distance comparable to that used for generation of PPP. A flow chart describing the centrifugation procedure is presented in Figure 1.

### Incubation of PRP with Native LDL or oxLDL

The fresh PRP samples were incubated without or with either oxLDL or native LDL (oxLDL, 2 mg/mL, cat# BT-910; nLDL, 5 mg/mL, cat# BT-903, Biomedical Technologies Inc., MA, USA) at increasing concentrations: 0-, 10-, 20-, and 40 μg/mL for 30 min at room temperature (RT). The oxLDL was generated by copper sulfate oxidation of LDL. The lot used in this study migrated 2.1-fold further than LDL on an agarose gel, and it was used for experiments within 4 weeks. The lowest concentration of 10 μg/mL corresponds to physiological levels found in individuals without metabolic syndrome (13). Plasma samples were further centrifuged, as described in Figure 1, and stored at −80°C until MV analysis.

### MV Labeling

Fifty microliters of freshly thawed plasma were transferred to a TruCount™ tube (BD Bioscience, NJ, USA) containing a lyophilized pellet, releasing a known number of fluorescent beads. MVs were then labeled with 5 μL fluorescein isothiocyanate (FITC)-conjugated lactadherin (83 μg/mL, Hematologic Technologies Inc., VT, USA). Lactadherin is a specific marker of MVs due to its binding to phosphatidyl-serin (PS) exposed on the surface of MVs (12). To identify MVs originating from platelets and whether or not they express CD36, MVs were further labeled with 3 μL allophycocyanin (APC)-conjugated antihuman CD41 (25 μg/mL IgG1, κ, clone HIP8, BioLegend, San Diego, CA, USA) and 3 μL phycoerythrin (PE)-conjugated antihuman CD36 (25 μg/mL IgG2a, κ, clone 5-27, BioLegend, San Diego, CA, USA). Isotype controls matching each antibody were used as negative controls, APC-conjugated mouse IgG1 κ (clone MOPC-21, BioLegend, San Diego, CA, USA) and PE-conjugated mouse IgG2a κ (clone MOPC-173, BioLegend, San Diego, CA, USA). After 30 min incubation time (4°C in the dark), 200 μL 0.22 μm filtered PBS was added to each labeled sample.

### Flow Cytometry

After incubation with antibodies and addition of PBS, the samples were immediately analyzed using BD FACSAria™ III High Speed Cell Sorter, which incorporates three air-cooled lasers at 375, 488, and 633 nm wavelengths and is equipped with BD FACSDiva™ software (v.6.1.3). The procedure was essentially as described in Ref. (12) except that no neutral density filter was used. The samples were analyzed at a max rate of 20000 events per second and when MV counts reached 500000 events (in two cases 1000000) within the MV gate, the analysis was stopped.

**Figure 2:**
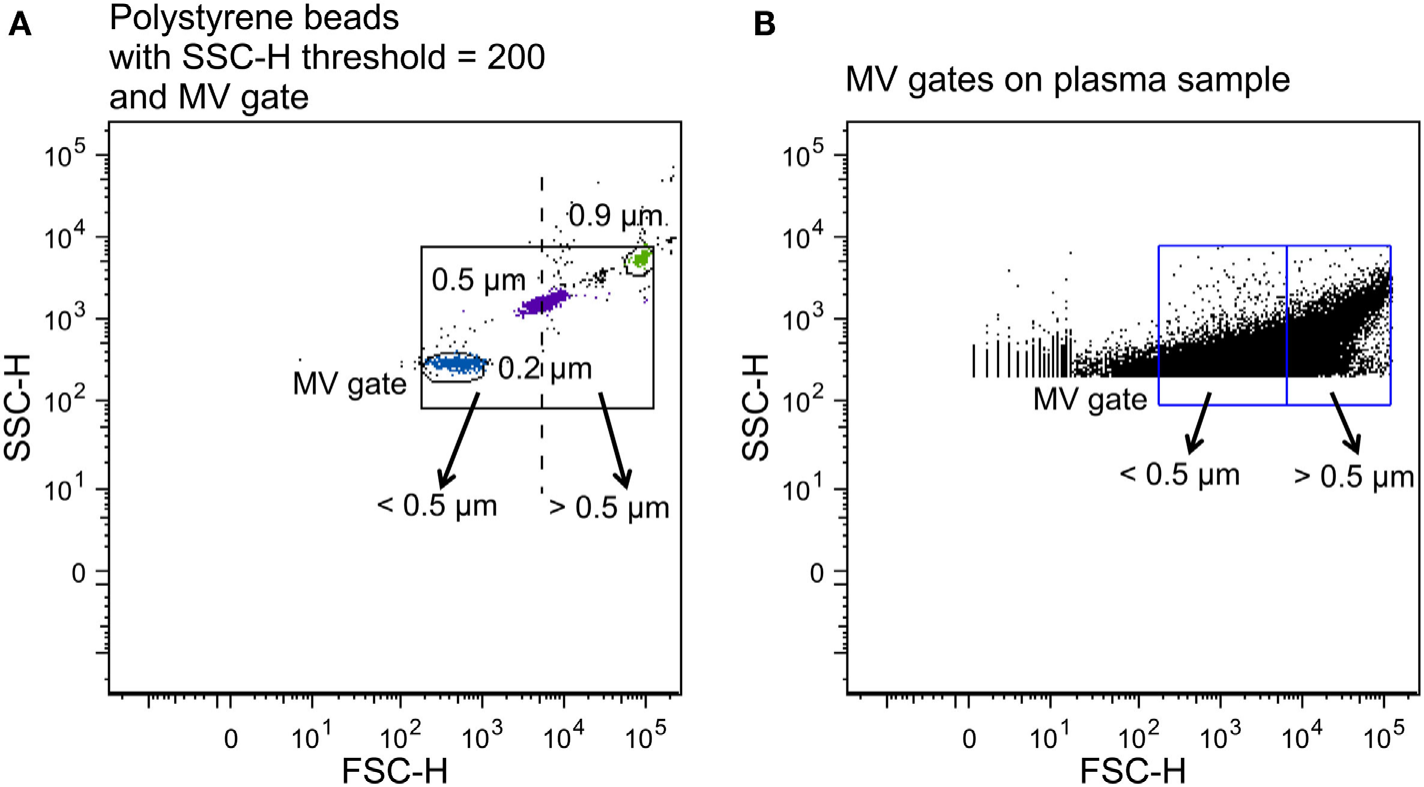
**Construction of MV gates using size-calibrated fluorescent beads**. **(A)** Selection of bead subsets using FACSAria instrument with a set SSC-H threshold of 200, and determination of small- (<0.5 μm) and large-size (>0.5 μm) gates. The solid square is the total MV gate and the dotted line separates the small- and the large-size gates. **(B)** Application of the MV gates to a plasma sample.

To establish a MV gate, preliminary standardization experiments were conducted using a blend of size-calibrated fluorescent beads with a size range of 0.2 (Invitrogen) to 3.0 μm [Megamix beads (0.5, 0.9, and 3.0 μm), Biocytex, Marseille, France]. The upper and outer limits of the MV gate were established just above the size distribution of the 0.9 μm beads, and the lower limit was set to include the 0.2 μm beads to obtain a MV gate of approximately 0.1–1 μm in a forward (FSC-H) and side scatter (SSC-H) setting (log scale) (Figure 2A) using the “auto-gate” function inside the Flow Jo™ software (v.8.8.7, Tree Star Inc., OR, USA). We defined MVs as particles within the MV gate, which had positive staining for lactadherin (PS^+^), and expressed cell-specific markers.

For MV quantification, TruCount™ beads (4.2 μm in diameter) with a known number of fluorescent beads were used according to the manufacturer’s manual. The following formula was used to calculate the number of MV events per microliter: (number of events in the MV region × number of beads per test)/(number of events in the absolute count bead region × test volume). The number of beads in TruCount™ tubes was provided by the manufacturer, and the test volume was 50 μL. PBS (0.22 μm filtered) was analyzed under conditions identical to the MV samples in order to obtain information of background noise.

To determine the fraction of small- and large-size MVs and investigate whether oxLDL affects the size distribution of MV release following incubation, two gates were established on the basis of the original MV gate. By using the 0.5 μm beads as the upper and lower limits of two new gates, we obtained a small-size gate ranging from the lower border of the 0.2 μm beads to the 0.5 μm beads and a large-size gate from 0.5 μm to the upper border of the 0.9 μm beads, in a FSC-H and SSC-H setting (Figure 2).

Mean fluorescence intensity (MFI) refers to the fluorescence intensity of each event in average and is, in our case, calculated using the geometric mean, which weights high and low values equally, and is applied when normal distribution is absent.

### Statistical Analysis

Statistical analyses were performed using SPSS Version 22 (SPSS Inc., IBM Corporation, NY, USA). MV data were expressed as median (interquartile range) and not normally distributed as evident by the Shapiro–Wilk test for normality. For analysis of two related samples, the Wilcoxon signed rank test was used, and for analysis of variance, the Friedman test was used. *P* values ≤0.05 indicated a statistically significant difference. For box plot, outliers were defined as cases that fall more than 1.5 times the box length from either end of the box.

## Results

### Study Participants

The study population consisted of healthy, lean males and females between 28 and 60 years of age. Characteristics of the study participants are given in Table 1.

**Table 1:** **Characterization of the study population (*n* = 11)**.

	Male (*n* = 7)	Female (*n* = 4)
Age (years)	40.86 ± 12.92	45.25 ± 8.73
BMI (kg/m^2^)	24.04 ± 3.06	23.89 ± 2.42
Hemoglobin (mmol/L)	9.24 ± 0.46	8.65 ± 0.69
Platelet count (10^9^/L)	263 ± 41.95	281.75 ± 77.94
Leukocyte count (10^9^/L)	5.17 ± 0.76	7.08 ± 2.16
Glucose (mmol/L)	4.83 ± 0.43	4.28 ± 0.69
ALT (U/L)	21.57 ± 5.65	14 ± 6.98
Total-C (mmol/L)	4.91 ± 1.18	4.73 ± 0.79
HDL-C (mmol/L)	1.33 ± 0.20	1.50 ± 0.27
LDL-C (mmol/L)	3.11 ± 1.11	2.80 ± 0.67
Triglycerides (mmol/L)	1.10 ± 0.12	0.93 ± 0.26

*Values are shown as mean ± SD. Abbreviations: BMI, body mass index; ALT, alanine aminotransferase; Total-C, total cholesterol; HDL-C, HDL cholesterol; LDL-C, LDL cholesterol*.

### The MV Profile of PRP and PFP After Incubation

To investigate the MV release from platelets when left for 30 min at RT, we compared MV counts and MFI values of PRP and PFP. The latter represent the MV profile at the time of blood collection.

**Figure 3:**
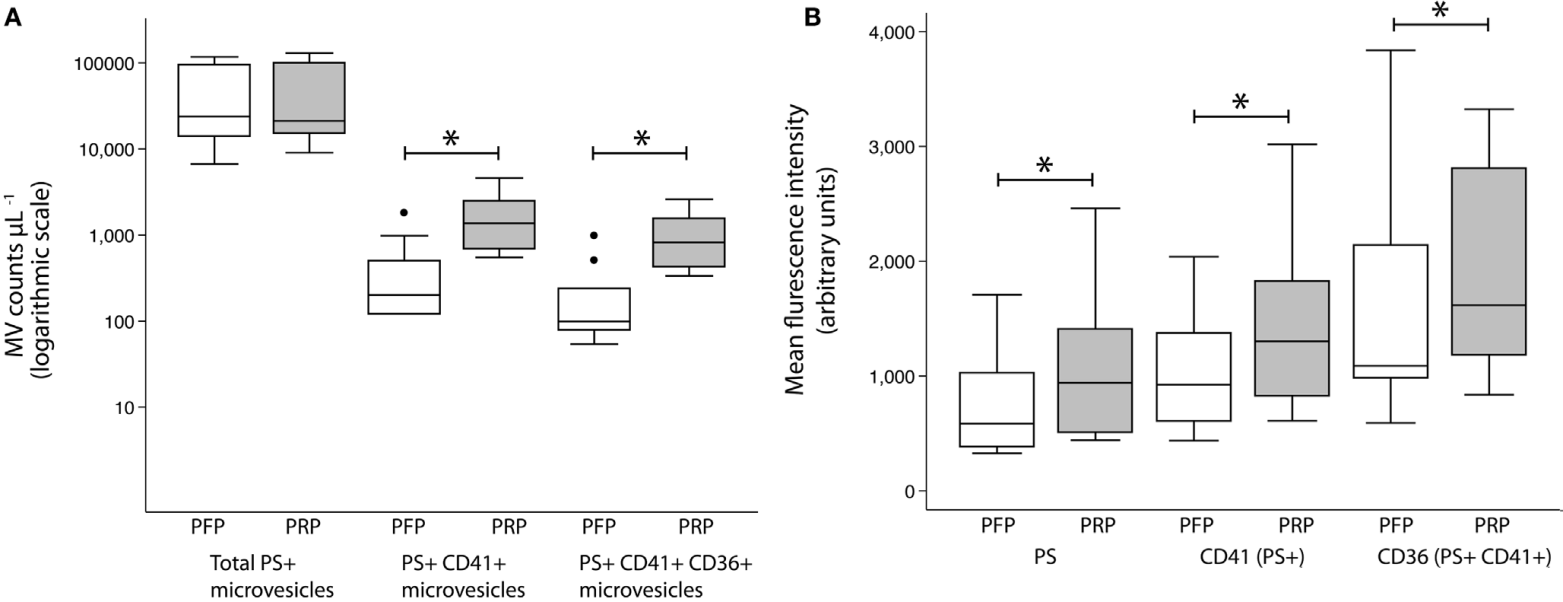
**MV release from PRP at room temperature**. Effect of incubation of PRP for 30 min at room temperature on MV counts **(A)** and MFI values **(B)** on MVs within the MV gate (total PS^+^ MVs) expressing CD41 (PS^+^CD41^+^ MVs) and CD36 (PS^+^CD41^+^CD36^+^ MVs) compared to MVs in PFP. Values are shown as counts per microliter plasma and expressed as median (interquartile range). *P < 0.005, Wilcoxon signed rank test. Circles represent outliers.

The CD41^+^ MV counts increased sixfold after 30 min at RT [200 (121–498) vs. 1374 (690–2499) counts per microliter plasma, *P* < 0.005]. Likewise, CD41^+^CD36^+^ MV counts increased eightfold [99 (79–239) vs. 819 (427–1552) counts per microliter plasma, *P* < 0.005]. No change in total MV counts was observed (Figure 3A). MFI values, representing the mean density of the antigen, were significantly higher in PRP compared with PFP, for lactadherin binding [585 (383–1028) vs. 941 (508–1410) counts per microliter plasma, *P* < 0.005] as well as CD41 [925 (608–1371) vs. 1303 (826–1824) counts per microliter plasma, *P* < 0.005] and CD36 [1089 (984–2139) vs. 1617 (1184–2811) counts per microliter plasma, *P* < 0.005] (Figure 3B).

### PMV Release After oxLDL Incubation

Platelet-rich plasma was incubated with oxLDL or nLDL as control in order to investigate the effect on platelet release of MVs, particularly platelet-derived CD36^+^ MVs. Table 2 shows CD41^+^ MV counts and CD41^+^CD36^+^ MV counts after incubation with oxLDL and control. To investigate whether incubations with oxLDL had any effect on the density of investigated MV labels, the MFI values of lactadherin binding, CD41, and CD36 were calculated within the MV gate.

**Table 2:** **MV counts within the MV gate**.

	CD41^+^	CD41^+^CD36^+^
**oxLDL**		
10 μg/mL	1129 (505–2837)	938 (260–1509)
20 μg/mL	1044 (565–2396)	867 (497–1471)
40 μg/mL	762 (620–2694)	695 (407–1695)
**nLDL**		
0 μg/mL	1374 (690–2499)	819 (427–1552)
10 μg/mL	699 (426–2084)	506 (240–1273)
20 μg/mL	1173 (696–2162)	819 (539–1433)
40 μg/mL	789 (533–3597)	710 (405–1878)

*Values are shown as counts per microliter plasma and expressed as median (interquartile range)*.

As shown in Table 3, the analysis of variance test showed no significant effect of any of the incubations for neither CD41^+^ nor CD41^+^CD36^+^ MV counts nor MFI values.

**Table 3:** **Analysis of variance for oxLDL and control incubations**.

	oxLDL	Control
**MV COUNT**		
CD41^+^ MV counts	0.427	0.301
CD41^+^CD36^+^ MV counts	0.792	0.409
**MFI**		
Lactadherin	0.465	0.525
CD41	0.591	0.591
CD36	0.288	0.167

**P* values for MV counts and MFI, determined by the Friedman test*.

### The Fraction of Small- Vs. Large-Size PMVs

**Figure 4:**
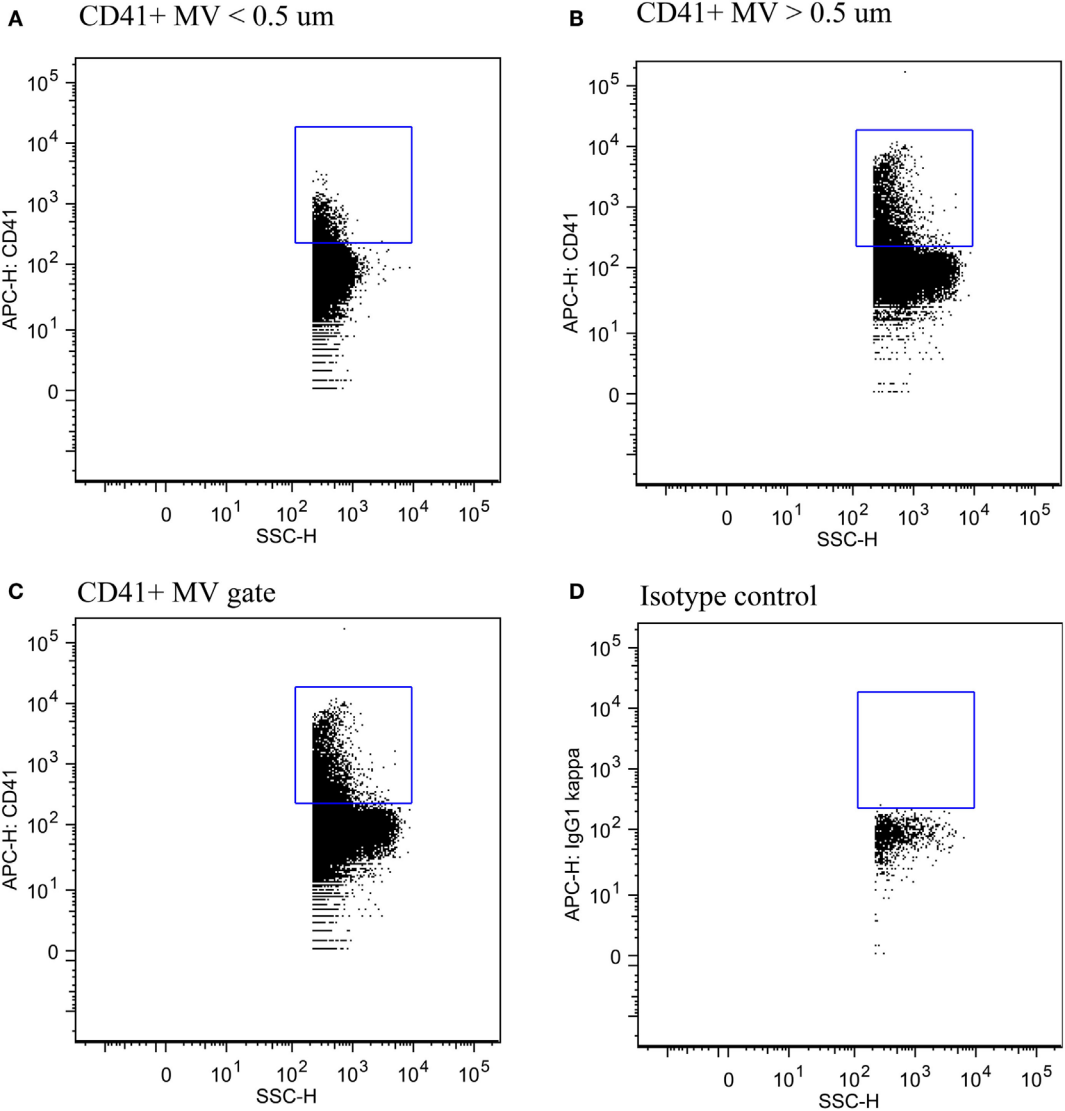
**Detection of PMVs**. **(A)** PMVs in the small-size gate, **(B)** the large-size gate, and **(C)** the total MV gate. **(D)** Isotype control showing no unspecific binding.

**Figure 5:**
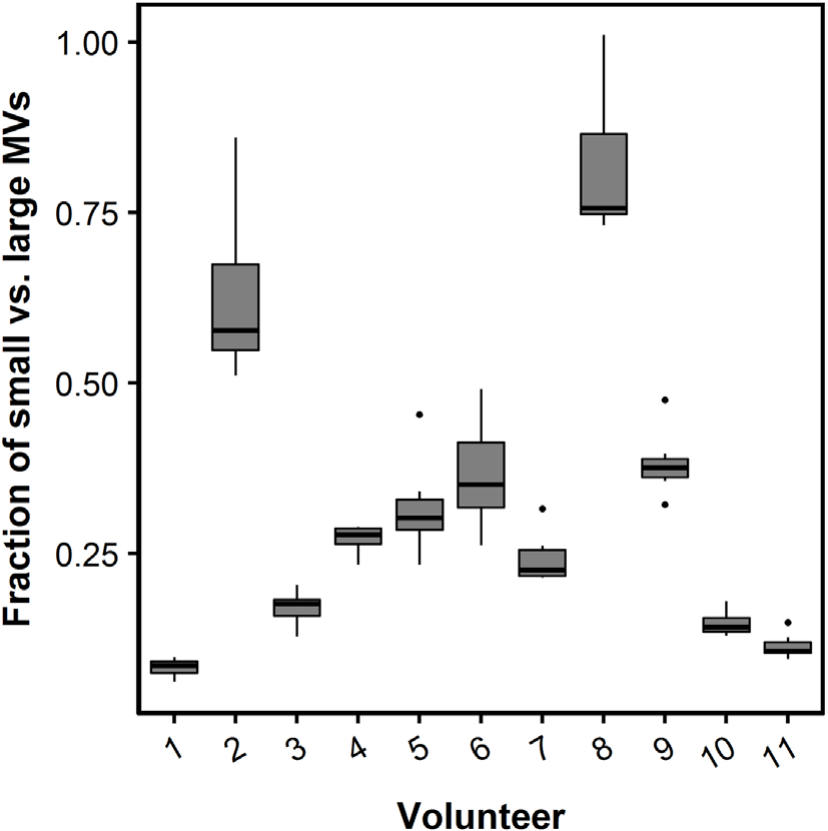
**Box plot of the fraction of small- and large-size MVs**. The relative contribution of small MVs (less than 0.5 μm in diameter) is presented as the fraction of small vs. large MVs. A fraction of one represents an equal number of small and large MVs. The fraction of small- vs. large-size MVs during each of the incubation conditions with nLDL and oxLDL are presented as box plots for each study participant. Boxes represent twenty-fifth to seventy-fifth quartile and median. Whiskers are maximum and minimum values. Circles represent outliers.

The fraction of small- vs. large-size PMVs was not affected when plasma was left for 30 min at RT [0.32 (0.18–0.50) vs. 0.29 (0.15–0.35), *P* = 0.113]. Figure 4 shows flow cytometric data of total PMV counts, small- and large-size PMV counts, as well as the isotype control, which show no unspecific binding. The intra- and interindividual differences between small- and large-size PMVs were explored using the described gating strategy. The fraction of small- and large-size PMVs was not significantly affected during incubation with oxLDL or nLDL, whereas the interindividual variation was 73% (Figure 5).

## Discussion

In this study, we investigated the MV profile after *in vitro* incubation of platelets with oxLDL, an interaction hypothesized to occur through the scavenger receptor CD36 located on the platelet surface. Furthermore, we compared the fraction of MVs larger than 0.5 μm as a measure of results obtained from conventional flow cytometers with MVs below the 0.5 μm limit. We did not observe any effect of the oxLDL stimulations on MV release, CD36^+^ MV release, antigen density, as evident by MFI values, or on the fraction of small- and large-size MVs. The fraction of small- and large-size MVs varies considerably between the individuals.

The activation of platelets plays a critical role in thrombosis. Platelet activation involves exposure of phosphatidylserine (PS) and subsequent MV release as reviewed in Ref. (14). Furthermore, the present results indicate that the density of both PS and cellular markers in MVs is increased upon platelet activation. PS is an important player in the coagulation cascade taking part in the assembly of coagulation complexes at the membrane surface, and their presence markedly accelerates the coagulation reaction (15). CD41 is located on the platelet surface. It is a receptor of fibrinogen involved in platelet aggregation, and upon platelet activation, additional complexes of CD41 are translocated to the membrane surface (16). Likewise, CD36 is upregulated at the platelet surface once the platelet is activated (17, 18). Oxidative stress is a central feature of atherothrombotic disease. Our study aimed to focus on the involvement of CD36 in the process of platelet stimulation and MV release induced by oxLDL. Surprisingly, in our set-up, oxLDL did not induce MV release. Other researchers have observed increased PMV release of 10–20% when adding similar oxLDL concentrations (19), as well as increased aggregation and an upregulated expression of P-selectin (19–21). The increased MV release was, however, based on incubation of washed platelets in buffer, and furthermore, MVs were quantified as total MFI in the MV gate as well as by absorbance of MV in an in-house ELISA Annexin V and CD41 antibodies. It could be argued that both these measures are indications of antigen density and not of MV number as presented in our study. Furthermore, the flow cytometric analyses were performed by a less sensitive flow cytometer, and the gating was set to exclude platelets at the upper border and with no indication of size. This gating strategy may include measurement of apoptotic bodies derived from platelets, whereas smaller MVs may not be detected. Thus, despite the previously demonstrated activation of platelets by oxLDL, there is no convincing evidence of MV release induced by oxLDL interaction with CD36 on platelets.

In Wang et al. (19), flow cytometry was performed by a FACS Calibur, which has a detection limit of around 0.5 μm using polystyrene beads as size calibrators (12). It is evident that the precise numbers of MVs are not comparable using less sensitive and more dedicated flow cytometers, but it is unknown whether the number of MVs larger than 0.5 μm correlates with total MV number.

By using the size-calibrated fluorescent beads with sizes of 0.2, 0.5, and 0.9 μm, we were able to establish a gate separating small-size from large-size MVs defined as below and above 0.5 μm, respectively. We observed a large interindividual difference in the fraction of small- and large-size MVs, in particular when compared to the variation seen within an individual during oxLDL and nLDL incubations. Given the fact that the study population was comprised of healthy individuals, this difference could be much larger when investigating patients (22). Our results indicate possible complications when comparing MV flow cytometric data analyzed on conventional flow cytometers which are not able to detect MVs below 0.5 μm to data analyzed on the more dedicated flow cytometers and stress the importance of standardization of flow cytometry MV quantification protocols.

One important preanalytical issue when quantifying plasma MVs derived from blood cells, in particular MVs from platelets, is the possible release of MVs during sampling and processing caused by a fall in temperature. We found a six to eightfold increase in two phenotypes of PMVs, in line with other investigators (23, 24). Whereas this finding stresses the importance of being careful when processing blood samples for PMV analyses, the fact that the total MV count was unaffected by this indicates that MVs may be studied on plasma where lesser care has been taken regarding temperature issues and centrifugation procedures during blood sample processing.

The strengths of our study include the assumption that we do not have any swarm detection when using this method and instrument, as previously described by our group (12). As for weaknesses of our study, the experimental set-up at RT may potentially influence platelet responsiveness to oxLDL. Future studies should include a positive control of platelet activation whose effect is documented by MV release. Furthermore, the oxLDL concentration interval may have been too narrow although it was chosen to be in the supraphysiological range and similar to that used in studies reporting on oxLDL-induced platelet activation (19–21).

## Conclusion

Incubation with oxLDL had no effect on release of MVs, and neither on the fraction between small- and large-size MVs. The CD41^+^ and CD41^+^CD36^+^ MV counts were increased in PRP when left for 30 min at RT, whereas total MV counts were unaffected. Furthermore, MFI values of lactadherin binding, CD41, and CD36, which were all increased on the surfaces of MVs from PRP, indicate a higher activity of platelets at RT. These findings address the importance of careful handling when investigating PMVs, but it might be possible to study total MV count when standard preanalytical procedures are not fully met. We found a large interindividual variation in the fraction of small- and large-size MVs, which is important to have in mind when comparing flow cytometry results analyzed on different flow cytometers.

We propose that the procoagulant activity and activation of platelets induced by interaction of platelet CD36 with oxLDL reported by others may not involve release of MVs. This proposal must, however, be confirmed in future studies. Furthermore, our results demonstrate great interindividual variability in size distribution of PMVs and thereby stress the importance for generation of standardized protocols for MV quantification by flow cytometry.

## Author Contributions

TN contributed to design of the study, carried out *in vitro* experiments, and was involved in acquisition of flow cytometry data. TN drafted the manuscript and approved the final version. MN contributed to design of flow cytometry set-up and performed the experiments. MN was involved in data analyses, revised the manuscript, and approved the final version. AH conceived the idea for the study and was involved in design and analyses of data. AH critically revised the draft manuscript and approved the final version. TN, MN, and AH all agree to be accountable for all aspects of the work in ensuring that questions related to the accuracy or integrity of any part of the work are appropriately investigated and resolved.

## Conflict of Interest Statement

The authors declare that the research was conducted in the absence of any commercial or financial relationships that could be construed as a potential conflict of interest.
